# User Experience of a Chatbot Questionnaire Versus a Regular Computer Questionnaire: Prospective Comparative Study

**DOI:** 10.2196/21982

**Published:** 2020-12-07

**Authors:** Mariska E te Pas, Werner G M M Rutten, R Arthur Bouwman, Marc P Buise

**Affiliations:** 1 Anesthesiology Department Catharina Hospital Eindhoven Netherlands; 2 Game Solutions Lab Eindhoven Netherlands; 3 Department of Electrical Engineering Eindhoven University of Technology Eindhoven Netherlands

**Keywords:** chatbot, user experience, questionnaires, response rates, value-based health care

## Abstract

**Background:**

Respondent engagement of questionnaires in health care is fundamental to ensure adequate response rates for the evaluation of services and quality of care. Conventional survey designs are often perceived as dull and unengaging, resulting in negative respondent behavior. It is necessary to make completing a questionnaire attractive and motivating.

**Objective:**

The aim of this study is to compare the user experience of a chatbot questionnaire, which mimics intelligent conversation, with a regular computer questionnaire.

**Methods:**

The research took place at the preoperative outpatient clinic. Patients completed both the standard computer questionnaire and the new chatbot questionnaire. Afterward, patients gave their feedback on both questionnaires by the User Experience Questionnaire, which consists of 26 terms to score.

**Results:**

The mean age of the 40 included patients (25 [63%] women) was 49 (SD 18-79) years; 46.73% (486/1040) of all terms were scored positive for the chatbot. Patients preferred the computer for 7.98% (83/1040) of the terms and for 47.88% (498/1040) of the terms there were no differences. Completion (mean time) of the computer questionnaire took 9.00 minutes by men (SD 2.72) and 7.72 minutes by women (SD 2.60; *P*=.148). For the chatbot, completion by men took 8.33 minutes (SD 2.99) and by women 7.36 minutes (SD 2.61; *P*=.287).

**Conclusions:**

Patients preferred the chatbot questionnaire over the computer questionnaire. Time to completion of both questionnaires did not differ, though the chatbot questionnaire on a tablet felt more rapid compared to the computer questionnaire. This is an important finding because it could lead to higher response rates and to qualitatively better responses in future questionnaires.

## Introduction

Questionnaires are routinely used in health care to obtain information from patients. Patients complete these questionnaires before and after a treatment, an intervention, or a hospital admission. Questionnaires are an important tool which provides patients the opportunity to voice their experience in a safe fashion. In turn, health care providers gather information that cannot be picked up in a physical examination. Through the use of patient-reported outcome measures (PROMs), the patient’s own perception is recorded, quantified, and compared to normative data in a large variety of domains such as quality of life, daily functioning, symptoms, and other aspects of their health and well-being [1,2]. To enable the usage of data delivered by the PROMs for the evaluation of services, quality of care, and also outcome for value-based health care correctly, respondent engagement is fundamental [3].

Subsequently, adequate response rates are needed for generalization of results. This implies that maximum response rates from questionnaires are desirable in order to ensure robust data. However, recent literature suggests that response rates of these PROMs are decreasing [4,5].

From previous studies, it is clear that factors which increase response rates include short questionnaires, incentives, personalization of questionnaires as well as repeat mailing strategies or telephone reminders [6-9]. Additionally, it seems that the design of the survey has an effect on response rates. Conventional survey designs are often perceived as dull and unengaging, resulting in negative respondent behavior such as speeding, random responding, premature termination, and lack of attention. An alternative to conventional survey designs is chatbots with implemented elements of gamification, which is defined as the application of game-design elements and game principles in nongame contexts [10].

A chatbot is a software application that can mimic intelligent conversation [11]. The assumption is that by bringing more fun and elements of gamification in a questionnaire, response rates will subsequently rise.

In a study comparing a web survey with a chatbot survey the conclusion was that the chatbot survey resulted in higher-quality data [12]. Patients may also feel that chatbots are safer interaction partners than human physicians and are willing to disclose more medical information and report more symptoms to chatbots [13,14].

In mental health, chatbots are already emerging as useful tools to provide psychological support to young adults undergoing cancer treatment [15]. However, literature investigating the effectiveness and acceptability of chatbot surveys in health care is limited. Because a chatbot is suitable to meet the aforementioned criteria to improve response rates of questionnaires, this prospective preliminary study will focus on the usage of a chatbot [13,16]. The aim of this study is to measure the user experience of a chatbot-based questionnaire at the preoperative outpatient clinic of the Anesthesiology Department (Catharina Hospital) in comparison with a regular computer questionnaire.

## Methods

### Recruitment

All patients scheduled for an operation who visit the outpatient clinic of the Anesthesiology Department (Catharina Hospital) complete a questionnaire about their health status. Afterward there is a preoperative intake consultation with a nurse or a doctor regarding the surgery, anesthesia, and risks related to their health status. The Medical Ethics Committee and the appropriate Institutional Review Board approved this study and the requirement for written informed consent was waived by the Institutional Review Board.

We performed a preliminary prospective cohort study and included 40 patients who visited the outpatient clinic between September 1, 2019, and October 31, 2019. Because of the lack of previous research on this topic and this is a preliminary study, we discussed the sample size (N=40) with the statistician of our hospital and this was determined to be clinically sufficient. Almost all patients could participate in the study. The exclusion criteria included patients under the age of 18, unable to speak Dutch, and those who were illiterate.

Patients were asked to participate in the study and were provided with information about the study if willing to participate. After permission for participation was obtained from the patient, the researcher administered the questionnaires. As mentioned above, informed consent was not required as patients were anonymous and no medical data were analyzed.

### The Two Questionnaires

**Figure 1:**
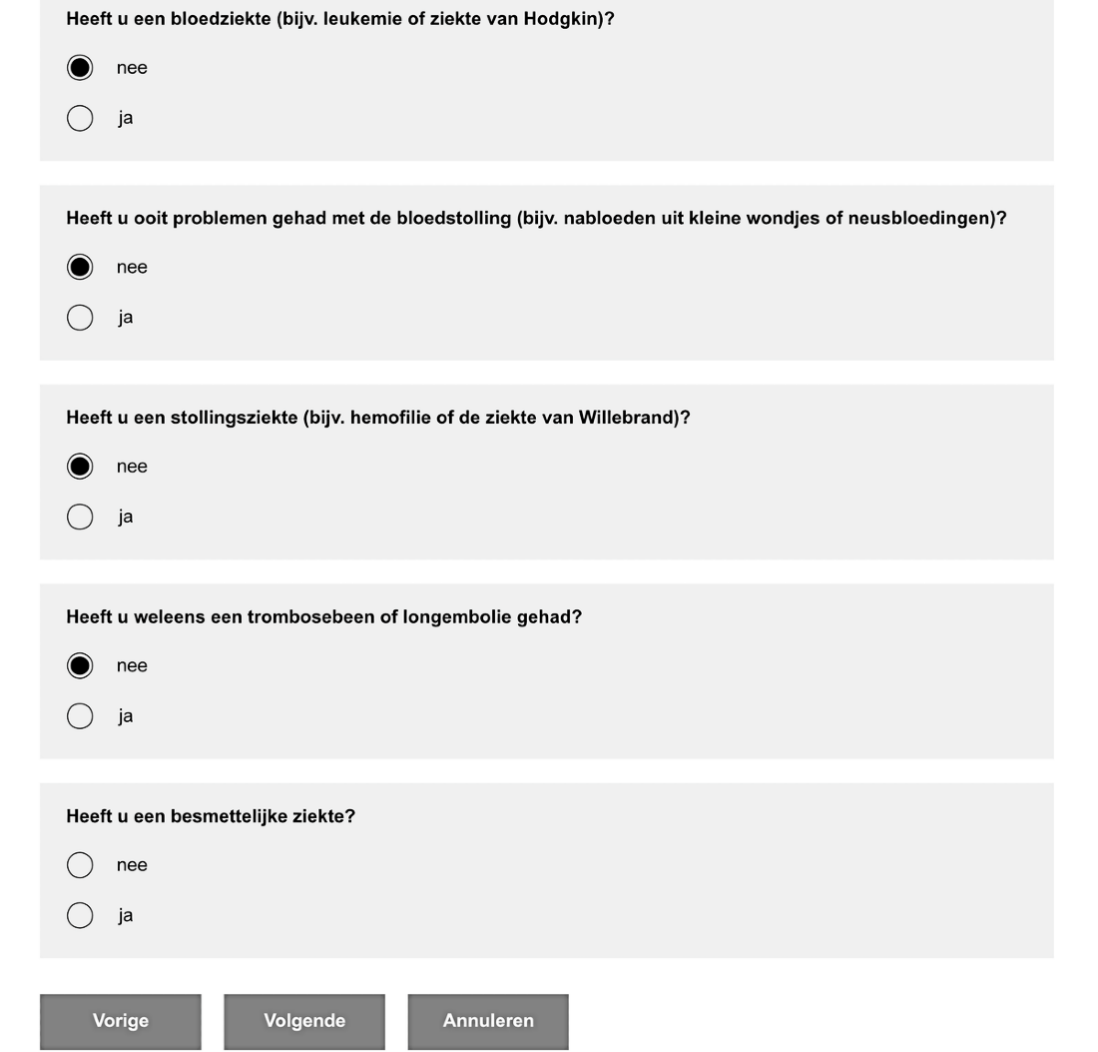
Computer questionnaire.

**Figure 2:**
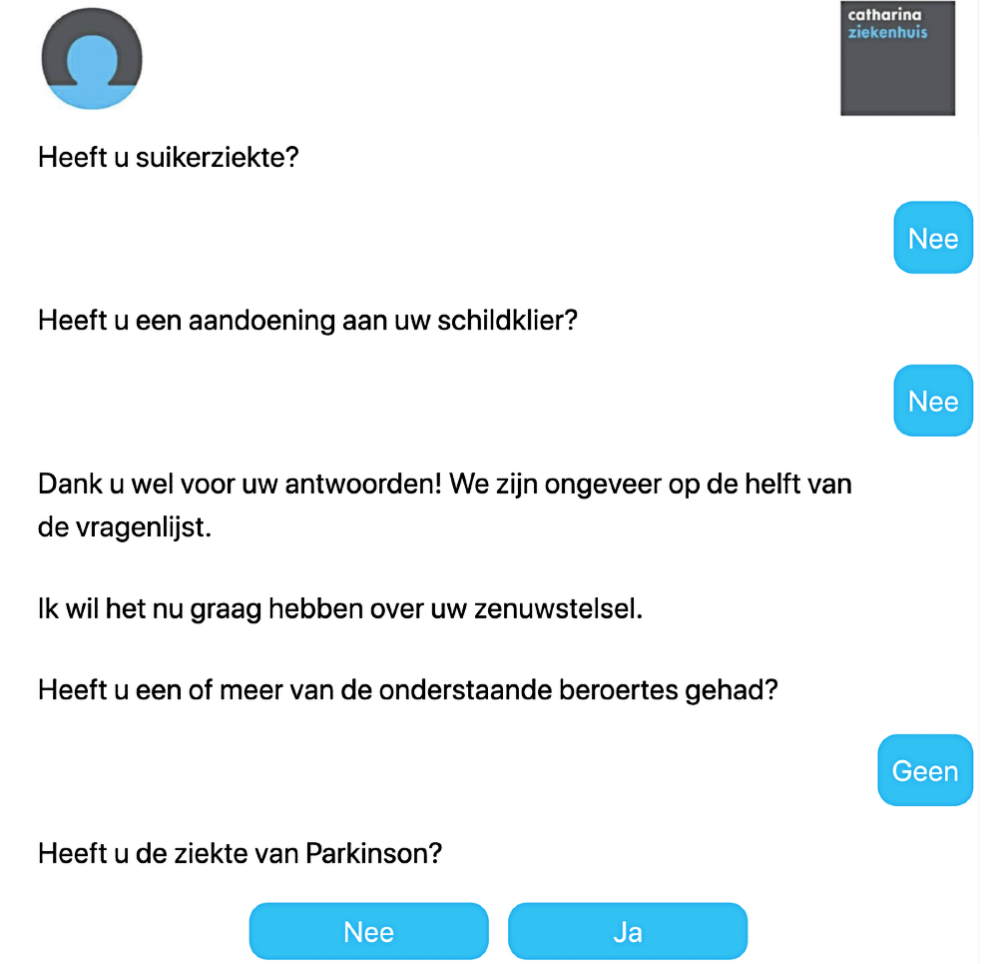
Chatbot questionnaire.

The computer questionnaire is the standard method at the Anesthesiology Outpatient Department (Figure 1). We developed a chatbot questionnaire (Figure 2) with identical questions to the computer version. This ensured that the questionnaires were of the same length, avoiding bias due to increased or decreased appreciation per question. The patients completed both the standard and chatbot questionnaires, as the standard computer questionnaire was required as part of the preoperative system in the hospital. Patients started alternately with either the chatbot or the computer questionnaire, in order to prevent bias in length of time and user experience. During the completion of both questionnaires, time required to complete was documented.

### The User Experience Questionnaire

**Figure 3:**
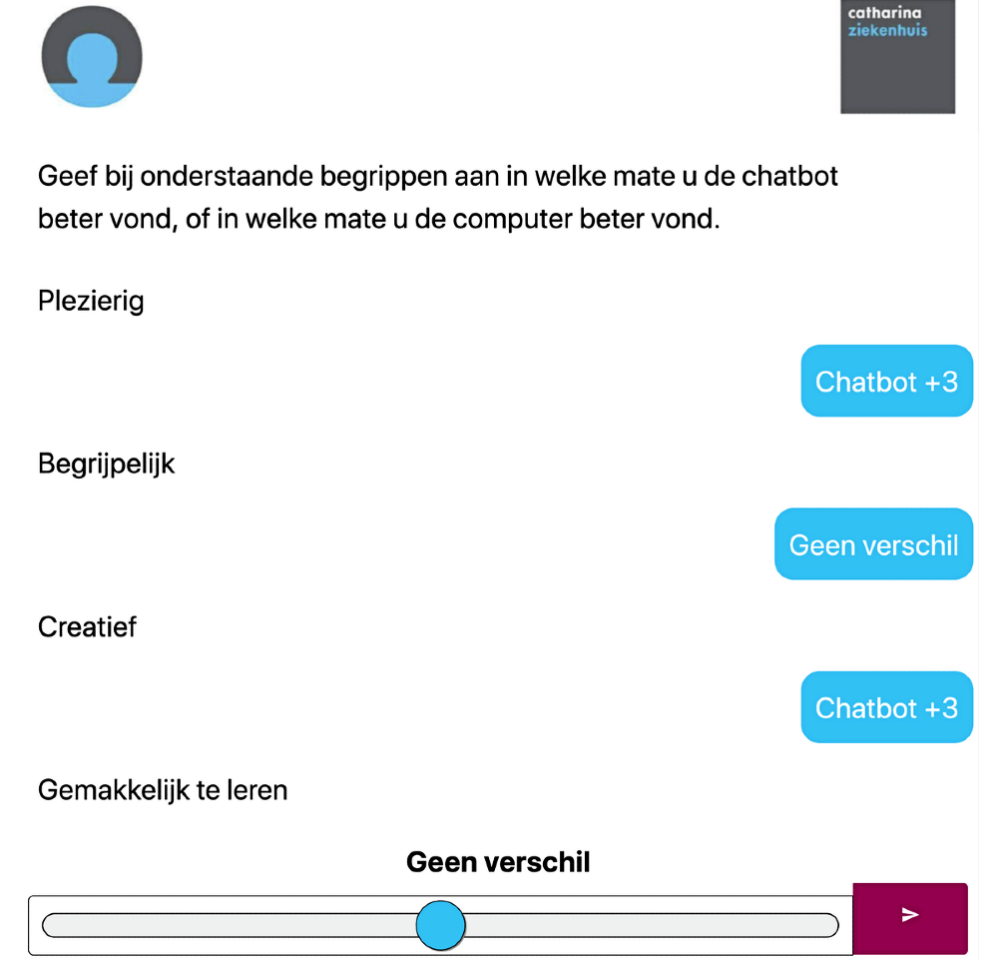
User Experience Questionnaire.

After completion of both questionnaires, patients provided feedback about the user experience. Patients were asked to rate their experience by providing scores for both questionnaires with the User Experience Questionnaire (UEQ; Figure 3). The reliability and validity of the UEQ scales were investigated in 11 usability tests which showed a sufficiently high reliability of the scales measured by Cronbach α [17-19]. Twenty-six terms were shown on a tablet and for each term patients gave their opinion by dragging the button to the “chatbot side” or to the “computer side.” They could choose to give 1, 2, 3, or 4 points to either the computer or the chatbot in relation to a specific term. If, according to the patient, there was no difference between the computer and the chatbot, he or she let the button in the middle of the bar.

The UEQ tested the following terms: pleasant, understandable, creative, easy to learn, valuable, annoying, interesting, predictable, rapid, original, obstructing, good, complex, repellent, new, unpleasant, familiar, motivating, as expected, efficient, clear, practical, messy, attractive, kind, and innovative.

As much as 20 of the 26 items were positive terms, such as “pleasant.” The other 6 are negative terms, such as “annoying.”

### Outcome Measures

The primary outcome measure of this research is the user experience score and the difference in score between the standard computer questionnaire and the chatbot questionnaire. Secondary outcome was duration to complete a questionnaire.

### Statistical Analysis

Data analysis primarily consisted of descriptive statistics and outcomes were mainly described in percentages or proportions. The unpaired *t* test was used to quantify significant differences between men and women and for time differences, because the data were normally distributed. A *P* value of .05 or less was chosen for statistical significance. Data were analyzed with SPSS statistics version 25 (IBM). Microsoft Excel version 16.1 was used for graphics.

This manuscript adheres to the applicable TREND guidelines [20].

## Results

The mean age of the 40 patients included, of whom 25 (63%) were women, was 49 (SD 18-79) years.

**Figure 4:**
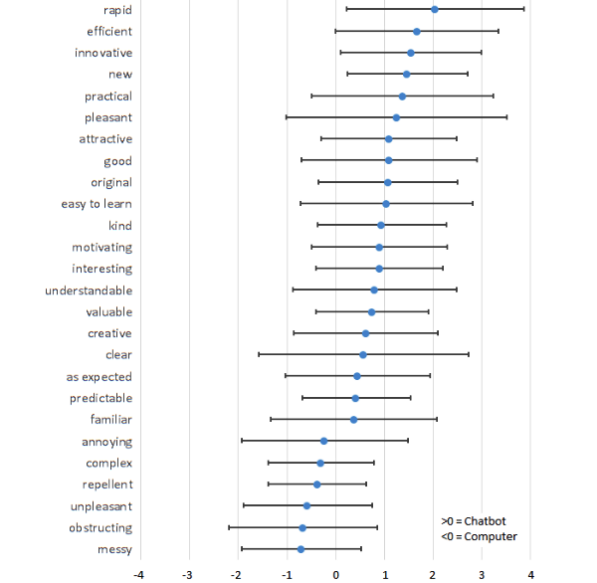
Average User Experience Questionnaire (UEQ) scores per term and standard deviation. A score above 0 illustrates that the term fits best with the chatbot. A score below 0 illustrates that the term fits best with the computer.

The average score per term was calculated and shown in Figure 4. The UEQ scores showed that patients favored the chatbot over the standard questionnaire. According to the graph, the patients prefer the chatbot for 20 of the 26 terms (77%), all of which are positive terms. The average values for the other 6 terms, which are the negative terms (23%), are shown to have a negative value. This indicates that on average the patients associated the standard questionnaire with negative terms.

In total, 1040 terms were scored. As much as 46.73% (n=486) of the user experience terms were scored positive for the chatbot, 47.88% (n=498) of the terms had preference neither for chatbot nor computer, and for 7.98% (n=83) of the terms patients preferred the computer.

Average time to completion of the computer questionnaire was 8.20 (SD 2.69) minutes; for the chatbot questionnaire this was 7.72 (SD 2.76) minutes. The questionnaire completed initially took on average more time to complete, as the data in Table 1 indicate.

Time to completion differed between men and women, but did not reach statistical significance. Every patient completed the second questionnaire statistically significantly faster than the initial one (chatbot *P*=.044, computer *P*=.012), irrespective of which questionnaire was completed initially (Table 1).

**Table 1:** Time to completion (minutes).

Criteria		Computer questionnaire completion time (minutes), mean (SD)	Chatbot questionnaire completion time (minutes), mean (SD)
**Average time to completion of computer- and chatbot-based questionnaire (n=40)**			
	All patients	8.20 (2.6)	7.72 (2.7)
**Average time to completion for men (n=15) versus women (n=25)**			
	Men	9.00 (2.7)	8.33 (2.9)
	Women	7.72 (2.6)	7.36 (2.6)
	*P* value	.148	.287
**Average time to completion depending on computer first (n=20) or chatbot first (n=20)**			
	Computer first	9.25 (2.4)	6.85 (2.1)
	Chatbot first	7.15 (2.6)	8.60 (3.0)
	*P* value	.012	.044

## Discussion

### Principal Findings

In this prospective observational study, we evaluated the user experience of a chatbot questionnaire and compared it to a standard computer questionnaire in an anesthesiology outpatient setting. Our results demonstrate that patients favored the chatbot questionnaire over the standard computer questionnaire according to the UEQ, which is in line with the previous research by Jain et al [21], who showed that users preferred chatbots as these provide a “human-like” natural language conversation.

Another intriguing result, as seen in Figure 4, is that the highest score to the chatbot was given for “rapid.” However, the time to completion of the questionnaires did not differ between the computer questionnaire and the chatbot questionnaire. This indicates that a questionnaire answered on a tablet may give the perception of being faster than a standard model answered on a computer. In addition, by using more capabilities of a chatbot it is possible to shorten the questionnaire, possibly leading to higher response rates, as mentioned by Nakash et al [6].

The second questionnaire took significantly less time to complete than the initial one, as the contents are identical between the 2 questionnaires. This is not an unexpected observation. Although time to completion of the initial questionnaire was significantly different compared to that of the second questionnaire, bias in the results was minimized by alternating the order of questionnaires.

### Comparison With Prior Work

Explanations for low response rates can be disinterest, lack of time, or inability to comprehend the questions. Furthermore, patient characteristics such as age, social economic status, relationship status, and those with preoperative comorbidities appear to have a negative influence on response rates, with the majority being nonmodifiable factors [22]. However, Ho et al [23] demonstrated that the method employed to invite and inform patients of the PROM collection, and the environment in which it is undertaken, significantly alters the response rate in the completion of PROMs. This means that, as expected in this study, there is a chance that response rates will rise by using a chatbot instead of a standard questionnaire.

### Gamification

As described in the study by Edwards et al [7], response rates will rise when incentives are used. Currently, questionnaires are often lacking elements motivating the patient to complete them. The introduction of nudging techniques, such as gamification, can help. Nudging is the subtle stimulation of someone to do something in a way that is gentle rather than forceful or direct, based on insights from behavioral psychology [24,25]. In a recent study by Warnock et al [26], where the strong positive impact of gamification on survey completion was demonstrated, respondents spent 20% more time on gamified questions than on questions without a gamified aspect, suggesting they gave thoughtful responses [26]. Gamification has been proposed to make online surveys more pleasant to complete and, consequently, to improve the quality of survey results [27,28].

### Limitations

There are some limitations to this research. First, as mentioned in the “Introduction” section, a chatbot can mimic intelligent conversation and is a form of gamification. In our study we had identical questionnaires and therefore did not explore how the chatbot could mimic intelligent conversation. However, this research demonstrates that only minor changes in the questionnaire’s design lead to improved user experience. Second, because both the tablet and the chatbot were different from the standard computer questionnaire, it is possible that the user experience was influenced by the use of a tablet rather than by the characteristics of a chatbot solely. Third, although the UEQ shows us that the patients appreciated the chatbot more than the computer, we did not use qualitative methods to understand what factors drove users to identify the chatbot as a more positive experience. Fourth, although we recommend the use of a chatbot in the health care setting to improve questionnaire response rate as seen in previous literature, we did not formally investigate this outcome.

### Future Research

Because patients preferred the chatbot questionnaire over the computer questionnaire, we expect that a chatbot questionnaire can result in higher response rates. This research is performed as a first step in the development of a tool by which we can achieve adequate response rates in questionnaires such as the PROMs. Further research is needed, however, to investigate whether response rates of a questionnaire will rise due to alteration of the design. In future research it will be interesting to investigate which elements of gamification are needed to have beneficial effects such as higher response rates and higher quality of the answers as well.

### Conclusions

Patients preferred the chatbot questionnaire over the conservative computer questionnaire. Time to completion of both questionnaires did not differ, though the chatbot questionnaire on a tablet felt more rapid compared to the computer questionnaire. Possibly, a gamified chatbot questionnaire could lead to higher response rates and to qualitatively better responses. The latter is important when outcomes are used for the evaluation of services, quality of care, and also outcome for value-based health care.

## Abbreviations

PROM: patient-reported outcome measure
UEQ: User Experience Questionnaire
